# Intestinal Biopsies for the Evaluation of Environmental Enteropathy and Environmental Enteric Dysfunction

**DOI:** 10.1093/infdis/jiab372

**Published:** 2021-07-17

**Authors:** Phoebe Hodges, Mizinga Tembo, Paul Kelly

**Affiliations:** 1 Tropical Gastroenterology and Nutrition Group, University of Zambia School of Medicine, Lusaka, Zambia; 2 Queen Mary University of London, London, United Kingdom

**Keywords:** environmental enteropathy, environmental enteric dysfunction

## Abstract

Environmental enteric dysfunction (EED) is a syndrome characterized by impairments of digestion and absorption and intestinal barrier failure in people living in insanitary or tropical environments. There is substantial evidence that it contributes to impaired linear growth of millions of children in low- and middle-income countries, to slowed neurocognitive development, and to diminished responses to oral vaccines. It represents the functional consequences of environmental enteropathy, an asymptomatic inflammatory disorder of the mucosa, and there is considerable overlap with the enteropathy observed in severe clinical malnutrition. The majority of studies of EED have employed functional tests based on lactulose permeation to define the presence of abnormal leak in the gut. However, where intestinal biopsies can safely be collected the opportunity then arises to study the underlying enteropathy in cellular and molecular detail, as well as to measure important functional elements such as enzyme expression. The purpose of this narrative review is to summarize the current understanding of environmental enteropathy obtained from small intestinal biopsies, and prospects for future work. We review histology, electron microscopy, transcription and protein expression, physiological measures, and the microbiome. We conclude that while noninvasive biomarkers of enteropathy and intestinal dysfunction permit large-scale studies of unquestionable value, intestinal biopsies are still required to investigate pathophysiology in depth.

Stunting in young children refers to attenuated linear growth [1]. It is a major problem globally [2], affecting 35% of children <5 years of age in Zambia, for example [3]. It is associated with increased mortality [4], impairment of neurocognitive development [5], and impaired responses to oral vaccines [6, 7]. There is now a large body of evidence showing that provision of extra nutrients does not correct linear growth faltering in low- and middle-income countries [8], even when given during early pregnancy [9], and environmental enteropathy (EE) is likely to be one of the major obstacles [10]. EE is characterized by intestinal inflammation, maldigestion and malabsorption, gut permeability, translocation of microbes or microbial products, and a systemic inflammatory response [11]. Importantly, EE is defined as an *asymptomatic* change in small intestinal structure and function, but there is considerable overlap between EE and the enteropathy associated with more severe forms of malnutrition, with or without persistent diarrhea.

The burden of infection with enteropathogens plays a major role in pathogenesis of stunting [12, 13]. There is now consistent evidence that children without diarrhea who have growth failure carry multiple pathogens. In Zambia the pathogen burden was markedly higher in children with stunting (mean, 4.7 per child) than those with good linear growth (mean, 2.2 per child) [14]. This is entirely consistent with the Etiology, Risk Factors and Interactions of Enteric Infections and Malnutrition and the Consequences for Child Health and Development (MAL-ED) study in 8 low- and middle-income countries [15] and a recent study in Bangladesh [16]. While improved water, sanitation, and hygiene (WASH) appears to be an obvious solution, the latest clinical trials in 3 countries indicate that improved WASH in the absence of nutritional intervention does not reduce stunting at all—at least using those WASH interventions that have been trialed [17]. These data suggest that WASH interventions need to have a more transformative effect on household hygiene [17]. Data from Zambia suggest that microbial translocation, a direct consequence of epithelial damage and mucosal barrier failure, diminishes with age during early childhood. We infer that EE is an adaptation to the continuing epithelial damage inflicted by unrelenting polymicrobial colonization by multiple pathogens [14]. Evidence for the central role of microbial translocation in driving inflammation and stunting was obtained from longitudinal studies in The Gambia [18].

## EVALUATION OF BIOPSIES USING THE DISSECTING MICROSCOPE

Tropical enteropathy was first described as a variation in villus morphology in Indian and African populations [19–22]. Initial reports described the fusion of finger-like villi into ridges and convolutions [23]. In a study of 3 jejunal biopsies taken on 476 occasions from 238 apparently healthy adults in a poor community in Lusaka, Zambia between 1999 and 2001, none showed finger-like villi [24]. This confirms that EE is virtually ubiquitous in this population, but the shape of villi conveys little additional information about pathophysiology or intestinal dysfunction. The extensive remodeling of the villi suggests that there are changes in expression of genes controlling morphogenesis and differentiation. Single-cell analysis of biopsies from Zambian adults indeed shows evidence of de-differentiation, including an increase in a population of surface mucosal cells that express a portfolio of mucus-associated genes and that have previously been observed in response to mucosal injury [25].

## EVALUATION OF BIOPSIES USING HISTOLOGY

**Figure 1.**
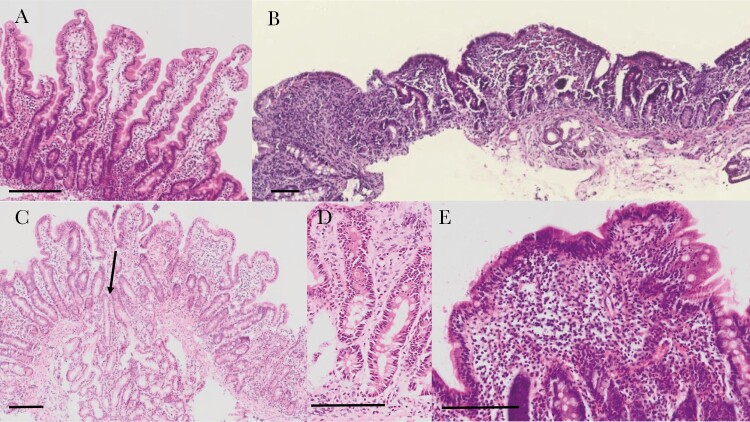
Histological sections of intestinal biopsies stained with hematoxylin and eosin. *A*, Long, slender villi in a well-orientated view as demonstrated by the crypts seen in longitudinal section. *B*, Severe enteropathy in a biopsy from a child with severe acute malnutrition complicated by human immunodeficiency virus infection and persistent diarrhea; there are no villi and severe inflammation of the lamina propria. *C*, Moderately inflamed section with shortened and widened villi visible, and with penetration of a Brunner gland into the mucosa (arrow). *D*, Section showing very long crypts and increased extracellular eosinophilic material from a biopsy showing subtotal villus atrophy. *E*, Lymphoid aggregate with marked intraepithelial lymphocytosis. Scale bars are 100 μm.

The great majority of histological analysis around the world relies on staining of formalin-fixed, paraffin-embedded (FFPE) tissue sections with hematoxylin and eosin (H&E) to highlight nuclear and cytoplasmic cellular features, respectively. H&E-stained FFPE sections reveal many important features of EE, including the remodeling of villi, enterocyte damage, reduction in secretory cells, and increases in lymphocyte populations in the epithelial and lamina propria compartments (Figure 1).

Early studies focused on the villus blunting, which is a prominent feature of EE [22]. This signifies both a reduction in villus height and an increase in villus width (Figure 1); the latter is probably attributable to an expansion of the mononuclear inflammatory cell infiltrate in the lamina propria. Studies in children in The Gambia defined the role of enteropathy in children with persistent diarrhea and malnutrition [26, 27]. Further studies included children with stunting without diarrhea [18], a clinical situation more like EE that strictly applies to asymptomatic children. These studies described a crypt hyperplastic enteropathy with Th1-like inflammation characterized by CD25^+^ lymphocytes and increased intraepithelial lymphocytes [18].

A recent study compared EE against biopsies from healthy American children and children with celiac disease [28]. EE was characterized by moderate villus atrophy that was not as severe as that seen in celiac disease. The intraepithelial CD3^+^ lymphocyte infiltration, however, was as severe as celiac, and the number of lymphoid aggregates was greater than celiac disease [28]. A study of enteropathy in Zambian children with severe acute malnutrition (SAM) showed severe pathology including total villus atrophy, especially in children with human immunodeficiency virus infection, associated with evidence of tight junction disorganization [29]. This was associated with evidence of microbial translocation and autoantibody formation, manifest as within-normal but elevated concentrations of celiac-like autoantibodies. Biopsies showed prominent epithelial damage, which would help explain the severity of microbial translocation [29].

Recently a scoring system for evaluation of EE in intestinal biopsies has been developed [30]. Using biopsies from 3 continents (Asia, Africa, and North America), a team of 3 pathologists scored biopsies from 45 children on 11 parameters. Features that appeared able to discriminate between healthy North American biopsies, celiac disease, and EE included severity of chronic inflammation, presence of intramucosal Brunner glands, intraepithelial lymphocyte infiltration, goblet cell depletion, Paneth cell depletion, enterocyte injury, epithelial detachment, and villous architecture. Further work is ongoing in a much larger study to refine this scoring system and compare geographical and other determinants of histological appearances. There is little doubt now that depletion of fully functional goblet cells and Paneth cells is a prominent feature of EE, and probably of crucial significance. Less is known about any changes in enteroendocrine and tuft cells, and more work is needed on this facet of the enteropathy.

## ELECTRON MICROSCOPY

**Figure 2.**
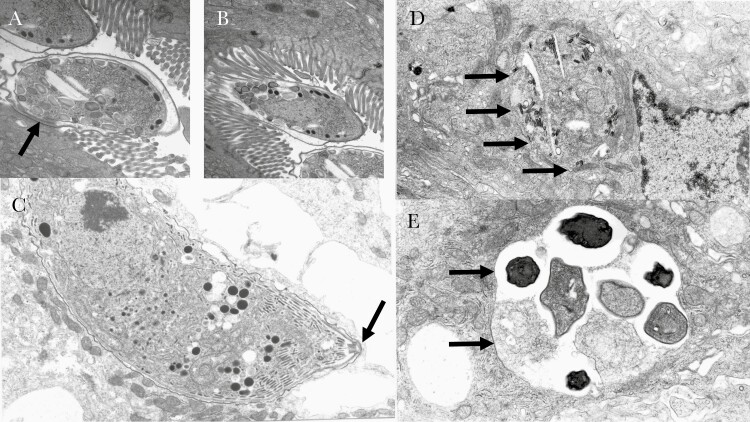
Electron microscopy of intestinal pathogens in small biopsies from AIDS patients in Zambia in the era before antiretroviral therapy. (*A*) and (*B*) fertilised macroamonts of *Cryptosporidium* spp which occupy an intracellular but extracytoplasmic niche, enclosed within plasma membrane and divided from the cytoplasm by the feeder organelle, an osmophilic band seen just beneath the parasite in (*A*) and indicated by an arrow. Obliteration of the brush border is evident across the area of attachment. (C) a trophozoite of *Cystisospora belli* (arrow) in a fully intracytoplasmic niche in an enterocyte, also from a Zambian AIDS patient. (*D*) *Enterocytozoon bieneusi*, and (E) *Encephalitozoon intestinalis*, both microsporidia which are now classified as fungi. Arrows indicate the boundary of the parasite.

Electron microscopy (EM) of tissues can take the form of scanning microscopy (which provides a view of a surface or interface such as freeze-fracture) or transmission EM (which images a cut section of tissue at high magnification and high resolution). Scanning EM of the small intestine shows villus morphology in a way analogous to the dissecting microscope, whereas transmission EM is more like a histological section. Transmission EM images of some important intestinal pathogens are shown in Figure 2. EM has not been applied to EE in asymptomatic children, but EM studies have shown a range of abnormalities in epithelial cells in biopsies from children with edematous SAM (also known as kwashiorkor) [31]. These include disorganization and shortening of the microvilli, abnormalities of nuclear morphology, abnormal mitochondria demonstrating swelling and disorganization of cristae, dilated endoplasmic reticulum, lysosomal inclusions, and reduced density of the basement membrane [31]. It is not known if these abnormalities are also features of EE, which is often associated with less severe forms of malnutrition; hence, more studies using EM would be very useful. Immunoelectron microscopy has been used to demonstrate the presence of brush border digestive enzymes in biopsies [32].

## PROTEIN CHARACTERIZATION: IMMUNOSTAINING, IMMUNOBLOTTING, AND PROTEOME

In the past, histochemistry using specific colorigenic substrates for each enzyme permitted localization of expression [20]. Such techniques were used to show reduction in brush border ATPase but preservation of alkaline phosphatase in patients with tropical sprue [20]. Protein expression can be quantified more precisely using Western blotting, or localized using immunohistochemistry or immunofluorescence. Veitch et al studied EE in Zambians compared to South Africans and identified T-cell activation using immunostaining for CD69 and HLA-DR [29, 33]. As described above, studies in The Gambia used immunostaining to demonstrate T-cell activation in children with malnutrition [18].

**Figure 3.**
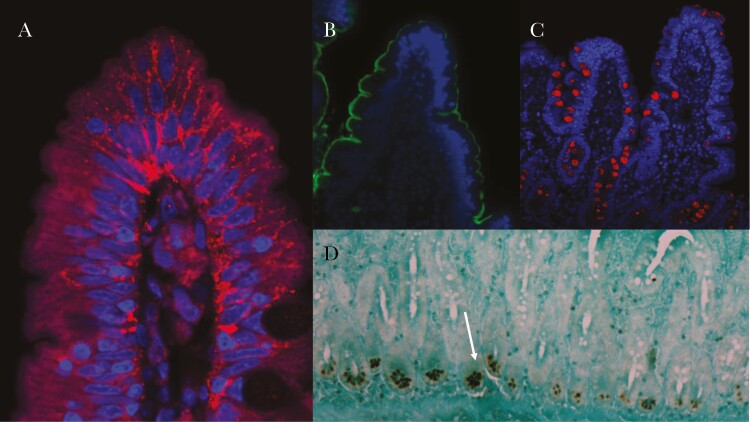
Examples of immunostaining of small intestinal biopsies from Zambian adult volunteers to show (*A*) claudin 4 disorganisation at the tip of a villus; claudin 4 is a tight junction protein, here stained in red, showing departure from the usual pattern of small foci of localisation near the brush border along the intercellular junction. (*B*) sucrase-isomaltase staining (in green) of the brush border around a villus. (*C*) staining of trefoil factor 3 (in red) which highlights goblet cells. (*D*) human defensin 5 staining of Paneth cells shown (arrow) using peroxidase immunohistochemistry. In *A-C* nuclei are stained in blue.

Immunohistochemistry for the human defensin 5 has been used to demonstrate that only Paneth cells synthesize this antimicrobial peptide (Figure 3), and it is clear that its expression is reduced in EE [34, 35].

The disadvantages of protein characterization using classical immunostaining are that it is at best semi-quantitative, and only 1 or 2 proteins can be imaged at a time. Western blotting improves on the quantitation that can be applied. Newer approaches to the second problem include multiplex immunostaining, which permits simultaneous imaging of 6 or more proteins, and flow cytometry on dissociated cell preparations, which can allow cellular expression of up to 40 proteins but at the cost of being unable to demonstrate anatomical localization. These techniques are of great interest to immunologists, but are only just beginning to be applied to EE as in a recent trial of amino acid and micronutrient supplementation [36]. Characterization of the proteome—the totality of all expressed proteins in a tissue or cell population—was used to characterize 2619 proteins in biopsies showing EE in Bangladeshi children with stunting [16]. The authors were able to identify modules of proteins that correlated with domains of pathophysiology in EE, including a module including proteins implicated in responses to infection (cathelicidin antimicrobial peptide, lipocalin 2, lactotransferrin, and resistin) associated with core taxa of an abnormal microbiota [16].

## TRANSCRIPTIONAL ANALYSIS

Assessment of gene expression in EE has been used for measurement of antimicrobial peptide messenger RNA (mRNA) using semi-quantitative reverse-transcription polymerase chain reaction and in situ hybridization [34, 35]. Compared to British adults, adults in Zambia had 1 order of magnitude less α-defensin mRNA. The astonishingly rapid development of nucleic acid sequencing technologies now permits RNA sequencing of tissues and even of individual cells. Several studies have now used transcriptomic profiling to develop an understanding of cellular changes in EE. Yu et al studied 259 children with EE in Malawi and defined severity using lactulose permeation [37]. They identified 51 genes whose expression was correlated with EE severity, including genes representing inflammation (especially interferon-mediated). Mucin gene expression was reduced. A study of Zambian adults used confocal laser endomicroscopy to study loss of epithelial integrity; 23 differentially expressed genes again included α-defensin and other antimicrobial peptide genes, inflammatory pathways, and genes related to goblet cell function [38]. An analysis of the transcriptome in 27 Zambian children with SAM again revealed similar themes [39]. Differentially expressed genes included genes for mucins and mucus integrity, antimicrobial defence, nutrient absorption, C-X-C chemokines, proteases, and anti-proteases. Phenotype–expression correlation analysis identified 1221 genes related to villus height, including increased cell cycling gene expression in more severe enteropathy. Amino acid transporters and zinc transporters were specifically increased in severe enteropathy, but transcripts for xenobiotic metabolizing enzymes were reduced [39]. In a study of Pakistani children with stunting, compared to controls in the United States, these themes were again recapitulated [40]. The transcriptome in the stunted children in Pakistan exhibited suppression of antioxidant, detoxification, and lipid metabolism genes, and induction of antimicrobial response, interferon, and lymphocyte activation genes [40]. This study also identified nicotinamide adenine dinucleotide phosphate (NADPH) oxidases as increased in EE, consistent with the study in Zambian children with SAM [39] and also observed in inflammatory bowel disease in children [41]. The single-cell RNA sequencing analysis previously referred to [25] identified many of the themes we can now regard as consistent across studies, and identified the cell subset that highly expresses NADPH oxidase genes as surface mucosal cells, which have previously been associated with mucosal injury [42]. However, the single-cell study found evidence of *reduced* cell proliferation in EE compared to American controls, in contrast to previous work [33]. The evidence for reduced cell cycling was strong, with consistently reduced expression of a whole module of mRNAs associated with cell turnover (*MKI67*, cyclins, Cyclin Dependent Kinases, topoisomerases, and polymerases) [25]. Although EE patients as a whole displayed lower levels of epithelial proliferation, more severe EE was associated with relatively higher epithelial proliferation than less severe EE. Single-cell analysis also corroborated the reductions in goblet cell numbers observed on histology. In summary, transcriptomic analysis of EE in several countries and diverse ages and clinical phenotypes allows several broad conclusions. Goblet cell numbers appear to be reduced, and there is strong evidence of impaired mucosal barrier function even while NADPH oxidase gene expression is increased. Expression of xenobiotic metabolizing genes is reduced. There is an increase in T-cell activation driving an inflammatory response dominated by all types of interferons. It should be noted, however, that the significance of some of these changes in mRNA needs to be clarified. For example, does upregulation of mucin genes signify an increase in mucins at the mucosal barrier, or is this a response to increased degradation? Careful analysis of the mucus layer, and goblet cells which secrete it, is urgently needed. Single-cell transcriptomic analysis now permits distinct cell lineages to be analyzed in unprecedented detail, and goblet cell physiology is an attractive target for such approaches.

## PHYSIOLOGICAL ANALYSIS

The use of intestinal biopsies to study digestive function dates back to the 1970s, long predating immunostaining or mRNA analysis [43]. Although physiologic analysis of intestinal biopsies has not been widely applied to the evaluation of EE, this approach has been used to demonstrate adaptation of the gut to various other conditions; for example, measurement of disaccharidase and aminopeptidase N activity in intestinal biopsies has been used to demonstrate colonic adaptation in children with short bowel syndrome [32]. In addition to demonstrating brush border enzymes on the apical membrane of colon enterocytes in these children through immunoelectron microscopy, stronger labeling for the enzymes aminopeptidase N, sucrase, and lactase phlorizin-hydrolase was also noted in the duodenal epithelium, suggesting that there is functional adaptation of the entire gut following resection. Intestinal adaptation is known to be regulated by enterohormones including glucagon-like peptide 2 which is expressed by L cells of the intestinal epithelium [44]. The enterotrophic properties of this hormone have led to its application as a therapeutic agent in patients with intestinal failure associated with short bowel syndrome who are dependent on parenteral nutrition (PN) [45], and its potential benefit in malnutrition enteropathy is also currently under evaluation [46], making it a target of interest for future physiological studies of EE.

Disaccharidase activity in duodenal biopsies has also been used as a measure of intestinal absorptive capacity in children with intestinal failure who have been receiving prolonged PN. Enzyme activities were determined by incubating biopsies with the relevant substrates for maltase, sucrase, and lactase and measuring production of glucose using the glucose oxidase method [47]. In PN-dependent patients, maltase and sucrase activities were consistently lower than in controls and patients on full enteral nutrition, although lactase activity was comparable. When patients were weaned off PN, disaccharidase activities recovered to control levels. It should be noted that duodenal mucosal inflammation was present in a subgroup of PN-dependent patients in this study, and that activity of sucrase, maltase, and lactase was lower in this group than in those without inflammation [48]. As chronic inflammation is also a feature of EE, albeit likely in a considerably different microbiological environment to PN-dependent children, this has implications for disaccharidase activity in EE. This has yet to be evaluated in EE using biopsies, although studies to validate the use of ^13^C-sucrose breath tests as a surrogate marker of sucrase activity in children with EE are in progress [49].

A study of disaccharidase activity in malnourished Brazilian infants found severely reduced levels of lactase mRNA in jejunal biopsies that could not be accounted for by villous atrophy alone. Sucrase mRNA levels were also reduced despite normal immunohistological staining for sucrase in villous enterocytes, suggesting a possible epigenetic mechanism for loss of disaccharidase activity in these children and further supporting a role for physiological assessment of biopsies in children with EE in addition to histological analysis [50].

Another potential use for intestinal biopsies, which has barely been exploited yet, is for the generation of organoids. Organoids are derived from stem cells and pluripotential cells, which allows for the study of differentiation and maturation. They can also be used to provide monolayers for the study of epithelial transport processes, which is potentially of enormous interest for understanding EE and other enteropathies. Recently a similar approach was used to study diarrhea induced by tyrosine kinase inhibitors [51].

## MICROBIOME ANALYSIS

While various studies have attempted to improve understanding of the role of the human gut microbiome in childhood malnutrition [16, 52, 53], these studies have not so far included the use of intestinal biopsy for characterization of the microbiome. There is a widely held belief that the mucosa-associated microbiome could be even more informative than analyzing fecal microbiota, and future studies will undoubtedly address this question directly.

## CONCLUSIONS

Environmental enteropathy is emerging as a major contributor to childhood malnutrition globally. As childhood malnutrition is proving very difficult to eradicate, and the world is set to fail to meet Sustainable Development Goals in the area of nutrition by 2030, the importance of EE is gaining increasing recognition. EE can be assessed using noninvasive techniques, but the information to be gained from judicious use of intestinal biopsies is irreplaceable. Recent transcriptomic analyses highlight the value of newer technologies in deepening our understanding of pathophysiology, and single-cell analysis is now taking off. Proteomic and immunostaining methods will now complement these and help open up novel therapeutic possibilities.

## Notes

***Financial support.*** This work was supported by the Bill & Melinda Gates Foundation (grant number OPP1066118).

***Potential conflicts of interest.*** All authors: No reported conflicts of interest.

Presented in part: 15th Asian Conference on Diarrhoeal Disease and Nutrition, Dhaka, Bangladesh, 28–30 January 2020.
